# Proteomic profiling of endometrioid endometrial cancer reveals differential expression of hormone receptors and MAPK signaling proteins in obese versus non-obese patients

**DOI:** 10.18632/oncotarget.22203

**Published:** 2017-10-31

**Authors:** Karen Klepsland Mauland, Zhenlin Ju, Ingvild Løberg Tangen, Anna Berg, Karl-Henning Kalland, Anne Margrete Øyan, Line Bjørge, Shannon N. Westin, Camilla Krakstad, Jone Trovik, Gordon B. Mills, Erling A. Hoivik, Henrica Maria Johanna Werner

**Affiliations:** ^1^ Centre for Cancer Biomarkers CCBIO, Department of Clinical Science (K2), University of Bergen, Bergen, Norway; ^2^ Department of Obstetrics and Gynecology, Haukeland University Hospital, Bergen, Norway; ^3^ Department of Bioinformatics and Computational Biology, The University of Texas M.D. Anderson Cancer Center, Houston, TX, USA; ^4^ Department of Microbiology, Haukeland University Hospital, Bergen, Norway; ^5^ Department of Gynecologic Oncology and Reproductive Medicine, University of Texas M.D. Anderson Cancer Center, Houston, TX, USA; ^6^ Centre for Cancer Biomarkers CCBIO, Department of Biomedicine, University of Bergen, Bergen, Norway; ^7^ Department of Systems Biology, The University of Texas M.D. Anderson Cancer Center, Houston, TX, USA

**Keywords:** endometrial cancer, obesity, biomarkers, hormone receptors, MAPK signaling

## Abstract

Endometrial cancer development is strongly linked to obesity, but knowledge regarding the influence of excess weight on endometrial tumor signaling pathways remains scarce. We therefore analyzed reverse phase protein array (RPPA) data for obesity-related protein expression patterns, using one training (n=272) and two test cohorts (n=68; n=178) of well-annotated samples from women treated for endometrioid endometrial cancer. Gene expression profiling and immunohistochemistry were used for cross-platform validation. Body mass index (BMI) was significantly correlated with progesterone receptor (PR) expression and a hormone receptor protein signature, across all cohorts. In two of the cohorts, BMI was negatively correlated with RTK- and MAPK-pathway activation, particularly phosphorylated MAPK T202 Y204 (p-MAPK) level. Using stepwise selection modelling, a BMI-associated protein signature, including phosphorylated estrogen receptor α S118 (p-ERα) and p-MAPK, was identified. In the subset of FIGO stage 1, grade 1-2 tumors, obese patients (BMI≥30) had better survival compared to non-obese patients in the two cohorts with longest follow-up time (p=0.042, p=0.058). Non-obese patients had higher p-MAPK levels, whereas obese patients had higher p-ERα levels and enrichment of gene signatures related to estrogen signaling, inflammation, immune signaling and hypoxia. In subgroup analysis of non-obese patients with FIGO stage 1 tumors, low PI3K-activation was associated with reduced survival (p=0.002, training cohort). In conclusion, increasing BMI is associated with increased PR and p-ERα levels and reduced MAPK signaling, both in all patients and in subsets with predicted excellent prognosis. The MAPK-pathway represents a potential therapeutic target in non-obese patients with low stage and low grade tumors.

## INTRODUCTION

Obesity is strongly associated with the development of endometrial cancer [1, 2], and it is estimated that around 30-40% of endometrial cancer cases in Europe can be attributed to obesity [3–5]. The World Health Organization (WHO) defines body mass index (BMI, kg/m^2^) from 18.5-24.9 as normal, 25.0-29.9 as overweight, and above 30 as obese [6]. The strong positive association between endometrial cancer incidence and BMI warrants further research to understand the mechanisms involved in this obesity-driven carcinogenesis, and implications for patient therapy.

So far, excess body weight is thought to influence endometrial cancer development through at least three different mechanisms; excess estrogen levels, insulin mediated effects, and secretion of pro-inflammatory mediators from the adipose tissue [7–10]. These systemic signals then mediate their cellular effects, among other, through activation of the estrogen receptor (ER) and receptor tyrosine kinases (RTKs), which induce activation of a range of intracellular signaling pathways, including transcription of ER target genes and increased phosphatidylinositide 3-kinase (PI3K)- and mitogen-activated protein kinase (MAPK) pathway signaling [8].

Most studies agree that increasing BMI is associated with characteristics of less aggressive endometrial cancer, such as low International Federation of Gynecology and Obstetrics (FIGO) stage, endometrioid histology and low histologic grade [11–15], but fewer studies have evaluated the impact of obesity on molecular tumor marker expression. Particularly, the PI3K pathway, one of the most frequently altered pathways in endometrial cancer, is thought to convey a range of obesity-mediated signals [16]. In a recent publication by Westin *et al.*, the effect of PTEN expression on patient survival was demonstrated to be dependent on obesity status, indicating that the effect of the genetic tumor make up may be dependent on the metabolic state [17]. Thus, new and interesting discoveries could be made by focusing on the potential impact of obesity on pathway activation and patient outcome using wide screen analyses for global protein and gene expression levels.

Reverse phase protein array (RPPA) is an antibody-based, proteomic method yielding quantitative and functional information on single proteins and pathways [18–20]. The included proteins cover major pathways of relevance to human cancer. Previous studies have shown great variability in correlation between mRNA levels, protein levels and functional protein levels [21, 22], underscoring that genetic and/or transcriptional analyses alone have limitations in predicting functionally relevant pathway aberrations and therapeutic targets in cancer.

In this study, we aimed to explore global protein expression patterns in primary endometrioid endometrial cancer (EEC) lesions in relation to BMI. As increasing BMI has been associated with development of low stage and low grade endometrioid tumors [11–15], we further wanted to study whether obesity is linked to survival and specific protein expression patterns in patient subgroups with presumed excellent prognosis. A training cohort and two test cohorts of well-annotated EEC patients with RPPA data were used. Overlapping global gene expression profiling and immunohistochemistry data were available for a subset of the patients.

## RESULTS

### BMI is correlated with hormone receptor expression and MAPK signaling in global protein expression analysis and by immunohistochemistry

**Figure 1:**
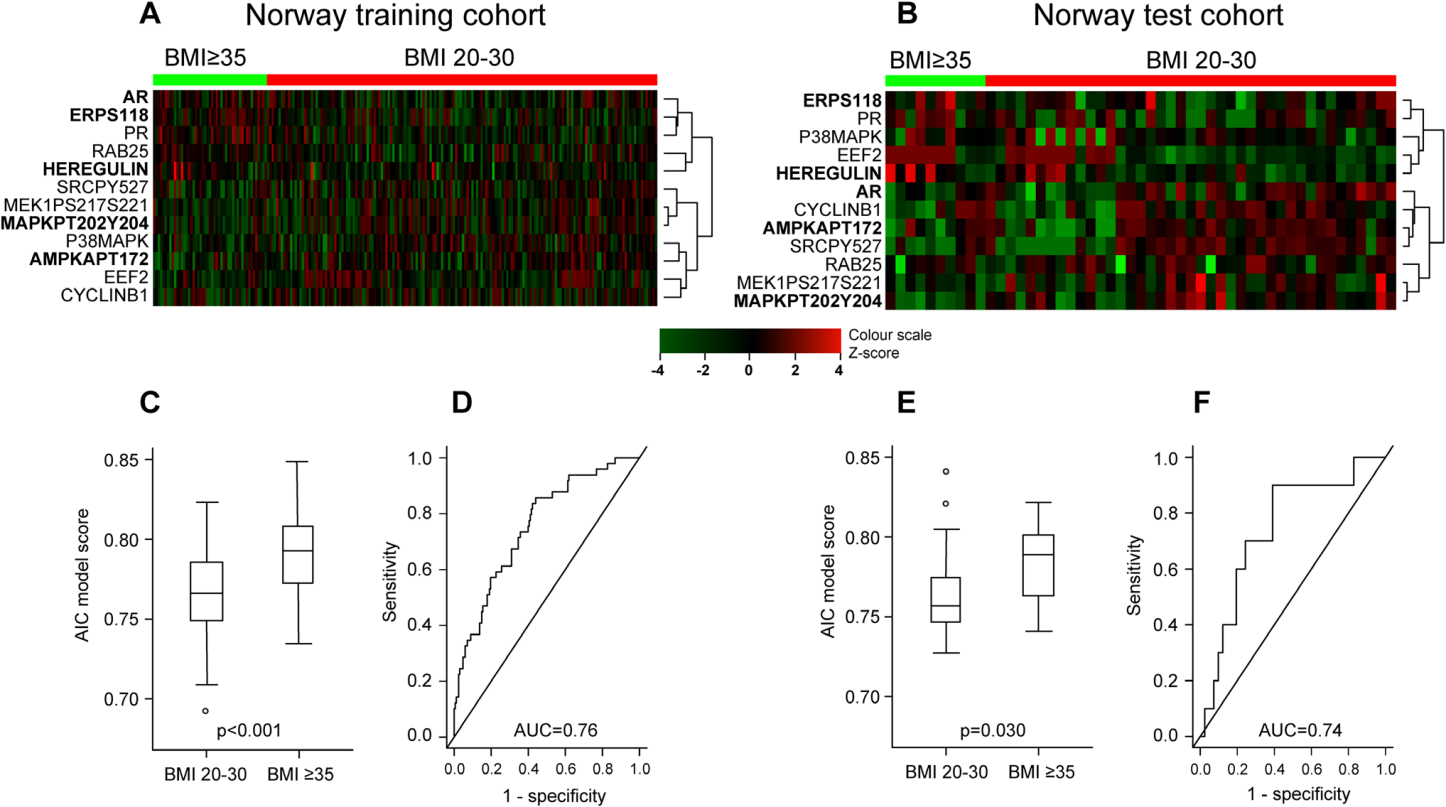
Differential protein expression pattern in endometrioid endometrial cancer patients with BMI20-30 versus BMI≥35 **(A)** Heatmap showing supervised hierarchical clustering of 12 proteins identified by LIMMA as differentially expressed in BMI groups 20-30 versus ≥35 in Norway training cohort (n=272). Protein names in bold were identified as the best predictors of BMI group by stepwise selection modelling using Akaike Information Criterion (AIC). **(B)** Supervised hierarchical clustering of the same 12 proteins in the Norway test cohort (n=68) revealed a similar pattern. **(C)** Distribution of AIC model score according to BMI groups in Norway training cohort. **(D)** Receiver operating characteristic (ROC) curve showing predictive value of the score with area under the curve (AUC) 0.76 in Norway test cohort. **(E)** Validation of model score distribution pattern in Norway test cohort. **(F)** Validation of ROC-curve in Norway test cohort.

Patient characteristics and comparison between the training cohort and the two test cohorts are given in Table 1 and Supplementary Table 1. First, we explored predefined pathway activation scores and their individual constituent proteins [18] (Supplementary Materials) against BMI as a continuous variable in the training set. Four predefined pathway activation scores showed a significant correlation with BMI (Table 2). We further tested the pathway scores and the individual constituents’ correlations with BMI in the test sets. In all three sets, increasing BMI was significantly correlated with increasing hormone receptor expression score and PR levels. Both Norwegian data sets showed signs of reduced activation of MAPK- and RTK-pathways with increasing BMI (Table 2), which was not confirmed in the MDACC test cohort. To further define the proteins that best characterized tumors arising in normal/overweight versus obese patients (i.e. a “BMI associated protein signature”), we divided BMI into two groups: BMI 20-30 and BMI≥35. LIMMA analysis (training set) revealed 12 proteins that were significantly differentially expressed between these two groups (unadjusted p-values <0.05), producing similar heatmaps for the two Norwegian data sets (Figure 1A and 1B). Using stepwise selection modelling (Akaike Information Criterion), a regression model was constructed from the training set that included the five proteins most predictive of BMI group (20-30 versus ≥35), defined by the following function: 1.19439 + 0.12406^*^p-ERα (S118) + 0.09128^*^AR - 0.08932^*^p-AMPK (T172) + 0.1622^*^HEREGULIN - 0.04781^*^p-MAPK (T202 Y204). The regression function was used to calculate a score which was significantly differentially distributed according to BMI groups, and the model predicted BMI groups with area under the curve (AUC) of 0.76 and 0.74, for the Norwegian training and test set, respectively (Figure 1C–1F). The model was not validated in the MDACC test set (Supplementary Figure 1). The score increased significantly when tested against BMI, categorized in 5-point intervals (also including the BMI 30-35 group) in the training set, and with a similar pattern although not significant in the Norwegian test set (Supplementary Figure 2). In line with the pathway analysis, increased hormone receptor expression in the high BMI category, and reduced expression of p-MAPK (T202 Y204) in the same group were observed using the model.

**Table 1:** Patient characteristics for Norwegian training and test cohorts used for RPPA analyses

	Norway training cohort (n=272) 2001 – 2013	Norway test cohort (n=68) 2011 – 2015	p-value^1^
	n (%)	n (%)	
**FIGO stage**			0.41
I	204 (75)	55 (81)	
II	24 (9)	5 (7)	
III	34 (13)	8 (12)	
IV	10 (4)	-	
**Grade**^2^			0.11
Grade 1-2	203 (76)	56 (85)	
Grade 3	65 (24)	10 (15)	
**Age (mean, SD)**	65.2 (11.5)	66.9 (10.9)	0.26
**Menopausal status**			0.05
Pre/perimenopausal	40 (15)	4 (6)	
Postmenopausal	232 (85)	64 (94)	
**BMI (mean, SD)**	29.4 (6.9)	29.1 (6.0)	0.73
**BMI groups**			0.90
20-25	84 (31)	20 (29)	
25-30	84 (31)	21 (31)	
30-35	55 (20)	17 (25)	
35-40	27 (10)	6 (9)	
≥40	22 (8)	4 (6)	

Abbreviations: BMI: Body mass index; FIGO: International Federation of Gynecology and Obstetrics; RPPA: Reverse phase protein array; SD: Standard deviation. n=number of patients.

^1^ P-value: Pearson Chi-square test for categorical variables, t-test for continuous variables.

^2^ Grade: missing for 4 tumors in Norway training cohort, missing for 2 tumors in Norway test cohort.

**Table 2:** RPPA pathway scores significantly correlated with BMI (Spearman, ρ), and their constituent proteins

Pathway score	Norway training cohort (n=272)		Norway test cohort (n=68)	
	ρ	p-value	ρ	p-value
**Hormone_a**^1^	0.23	<0.001	0.25	0.038
**p-ERα (S118)**	0.25	<0.001	0.29	0.015
ER	0.08	NS	0.12	NS
**PR**^2^	0.24	<0.001	0.42	<0.001
AR	0.17	0.004	-0.07	NS
Hormone_b	0.15	0.015	-0.05	NS
INPP4B	0.19	0.002	-0.15	NS
GATA3	0.11	0.08	0.02	NS
BCL2	0.04	NS	0.07	NS
**RAS_MAPK**	-0.13	0.034	-0.26	0.034
p-CJUN (S73)	-0.02	NS	-0.09	NS
p-CRAF (S338)	0.03	NS	-0.31	0.009
p-JNK (T183 Y185)	-0.11	0.07	-0.08	NS
**p-MAPK (T202 Y204)**	-0.20	0.001	-0.30	0.012
p-MEK1 (S217 S221)	-0.18	0.003	-0.19	NS
p-P38 (T180 Y182)	-0.09	NS	-0.14	NS
p-YB1 (S102)	-0.02	NS	0.11	NS
RTK	-0.16	0.008	-0.20	0.09
p-EGFR (Y1173)	-0.01	NS	-0.01	NS
p-HER2 (Y1248)	-0.07	NS	-0.07	NS
p-HER3 (Y1289)	-0.00	NS	-0.05	NS
p-SHC (Y317)	-0.16	0.010	-0.06	NS
p-SRC (Y416)	-0.11	0.07	-0.27	0.024
**p-SRC (Y527)**	-0.18	0.003	-0.26	0.031

Abbreviations: BMI: body mass index; NS: not significant; RPPA: Reverse phase protein array; ρ: Spearman correlation coefficient. p-values <0.1 are shown. Pathways and single protein constituents that were correlated with BMI in both data sets are marked in bold. p- indicates phospho-protein (site of phosphorylation in parenthesis).

^1^: Hormone receptor score also significantly correlated with BMI in MDACC data set.

^2^: PR expression also significantly correlated with BMI in MDACC data set.

We further compared expression level of selected RPPA proteins and phospo-proteins with mRNA expression and immunohistochemistry (IHC) data (Table 3). RPPA data correlated significantly with mRNA and IHC data for hormone receptors (all p-values <0.001), but stronger correlations were seen for total ERα compared to p-ERα (S118). The expression levels of MAPK pathway score proteins, which were all phospho-proteins, showed much weaker correlations with mRNA levels. As expected, we noted stronger correlations with mRNA for total-protein (tested where available, Table 3). A significant correlation was found between BMI and mRNA level for *PGR* in both Norwegian cohorts (ρ=0.24, p=0.007 training cohort, ρ=0.32, p=0.019 test cohort). Also, by IHC, PR loss was more common in non-obese patients (p=0.015 and p=0.007 for Norway training and test cohorts, respectively, Pearson Chi-Square test). No similar trend was found for *ESR1/*ERα or *AR/*AR levels by mRNA or IHC (data not shown).

**Table 3:** Correlations (Spearman, ρ) between RPPA and mRNA expression levels in Norway training set for hormone receptor- and MAPK activation score proteins (total protein included where available)

Protein (RPPA/IHC)	Gene	Agilent Probe	ρ (mRNA)	p-value	ρ (IHC)	p-value
ERα	*ESR1*	A_23_P309739	0.76	<0.001	0.47	<0.001
p-ERα (S118)	*ESR1*	A_23_P309739	0.53	<0.001	0.23	<0.001
PR	*PGR*	A_23_P138938	0.55	<0.001	0.48	<0.001
AR	*AR*	A_23_P113111	0.72	<0.001	0.48	<0.001
p-MAPK (T202 Y204)	*MAPK1*	A_24_P237265	0.16	0.06		
p-MAPK (T202 Y204)	*MAPK3*	A_23_P37910	0.18	0.04		
p-MEK1 (S217 S221)	*MAP2K1*	A_23_P20248	0.16	0.08		
p-MEK1 (S217 S221)	*MAP2K2*	A_23_P208835	-0.15	0.09		
MEK1	*MAP2K1*	A_23_P20248	0.27	0.002		
p-JNK (T183 Y185)	*MAPK8*	A_24_P286898	0.07	0.41		
p-P38 (T180 Y182)	*MAPK14*	A_23_P426292	0.15	0.09		
P38	*MAPK14*	A_23_P426292	0.41	<0.001		
p-CJUN (S73)	*JUN*	A_23_P201538	0.02	0.86		
p-CRAF (S338)	*RAF1*	A_23_P40952	0.06	0.47		
CRAF	*RAF1*	A_23_P40952	0.20	0.02		
p-YB1 (S102)	*YBX1*	A_32_P218989	-0.23	0.01		

Correlations between RPPA expression level and IHC expression (SI) shown for hormone receptors.

Abbreviations: IHC: Immunohistochemistry; ρ: Spearman correlation coefficient; RPPA: Reverse phase protein array; SI: Staining index. p- indicates phospho-protein (site of phosphorylation in parenthesis).

### Obese patients with FIGO stage 1, low grade tumors have better survival compared to non-obese patients

**Figure 2:**
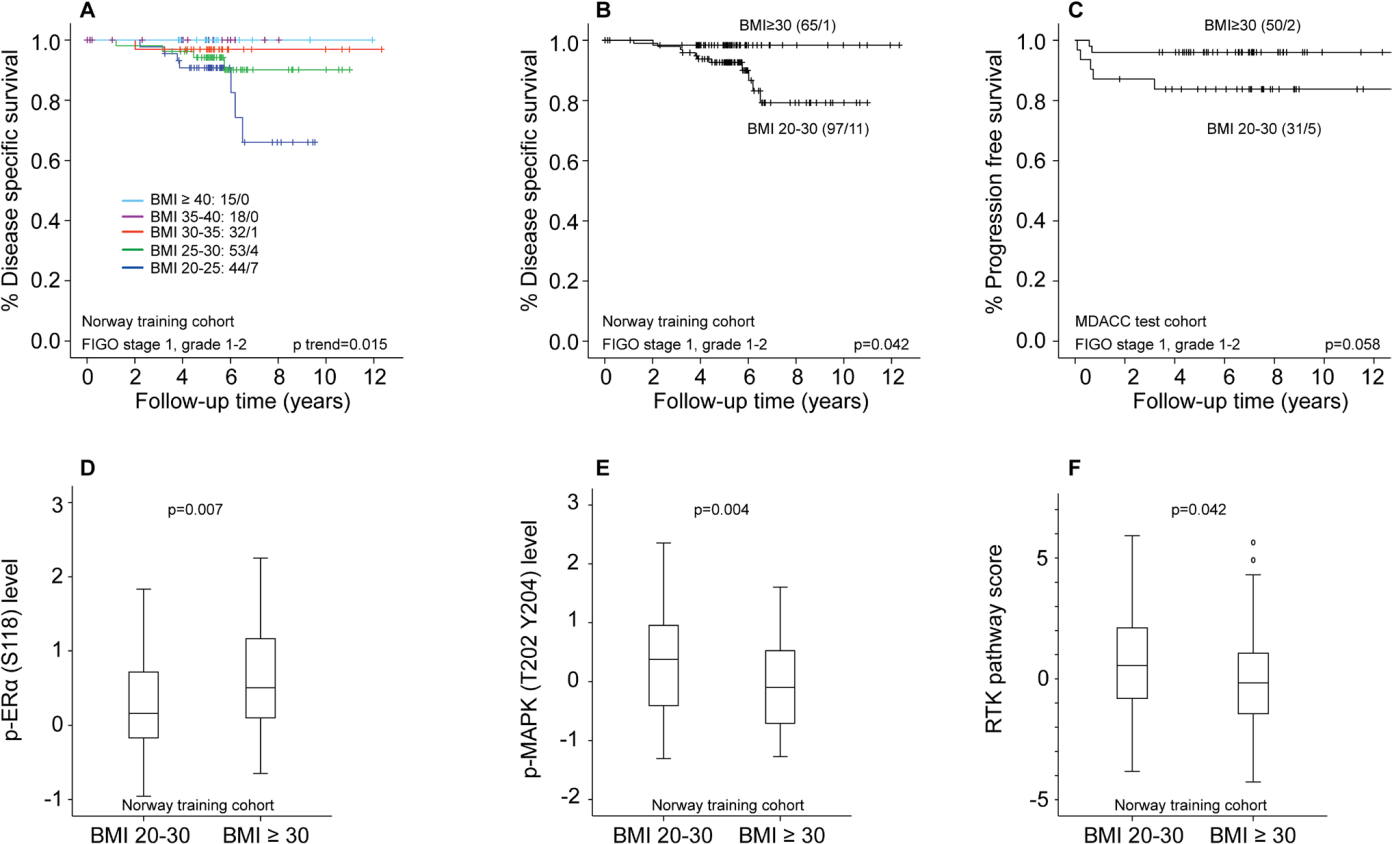
Differential survival and protein expression in obese versus non-obese endometrioid endometrial cancer patients with FIGO stage 1, grade 1-2 tumors **(A)** Disease specific survival (DSS) according to WHO BMI categories in patients with FIGO stage 1, grade 1-2 tumors. **(B)** DSS according to BMI categorized as non-obese (BMI20-30) and obese (BMI≥30) in FIGO1 grade 1-2 tumors. **(C)** Progression free survival according to BMI categorized as non-obese (BMI20-30) and obese (BMI≥30) in FIGO1 grade 1-2 tumors. **(D)** Significant differential distribution of p-ERα (S118) level in non-obese and obese patients. **(E)** Significant differential distribution of p-MAPK (T202 Y204) level in non-obese and obese patients. **(F)** Significant differential distribution of RTK pathway score in non-obese and obese patients.

We further examined the prognostic impact of BMI on disease-specific survival (DSS) in patients with FIGO stage 1, grade 1-2 tumors (training set, n=162). When grouping BMI in categories of 5 (WHO BMI classification), patients with BMI in the higher categories had markedly better survival compared to patients with lower BMI (p_trend_=0.015, Figure 2A). Applying BMI≥30 as cut-off for obesity (WHO criteria), obese patients had significantly better DSS compared to the normal/overweight group (p=0.042, Figure 2B). A similar pattern, with a tendency to improved survival for the obese patients, was noted in the MDACC test cohort, using progression free survival (PFS) as outcome measure (p=0.058, Figure 2C). However, this difference was not seen in the Norwegian test cohort. Of note, the events in the training set occurred late: most endometrial cancer deaths were observed after five years of follow-up (Figure 2A and 2B). The median time to recurrence was 1.8 years. In a multivariable Cox analysis including BMI (continuous variable), myometrial invasion (MI) and age (continuous variable), BMI and MI were both borderline significant predictors of survival (BMI HR 0.88, 95% CI 0.76 – 1.01, p=0.063 and MI≥50% HR 3.15, 95% CI 0.92 – 10.74, p=0.067), whereas age was not (HR 1.03, CI 0.97 – 1.09, p=0.35).

### Differential protein and gene expression patterns in FIGO stage 1, low grade tumors according to BMI groups

We assessed differential protein expression pattern and pathway activation scores in non-obese (BMI20-30) and obese (BMI≥30) patients with FIGO stage 1, grade 1-2 tumors. In the training cohort, non-obese patients had lower expression of p-ERα (S118) compared to obese patients (p=0.007, Figure 2D). In addition, they had higher expression of proteins indicating MAPK pathway activation: p-MAPK (T202 Y204), p-JNK (T183 Y185), p-MEK1 (S217 S221) and P38 (Figure 2E, Supplementary Table 2), as well as increased RTK-activation, (p=0.042, Figure 2F, all raw p-values). We performed the same analysis on patients with FIGO stage 1, ERα positive tumors by IHC, to a large extent overlapping with the grade 1-2 tumors. A similar survival curve and protein expression pattern was seen here (Supplementary Figure 3). In analyses of both test sets, p-MAPK (T202 Y204) was the most significantly differentially expressed protein between obese and non-obese patients with FIGO stage 1, grade 1-2 tumors (p=0.006 in Norway test set, p=0.016 in MDACC test set). p-ERα (S118) and RTK expression did not differ between these groups in any of the test sets. p-MAPK (T202 Y204) level was not associated with survival in the studied cohorts (analyzed as a continuous variable in univariable Cox-models, data not shown).

To further explore transcriptional alterations between tumors arising in obese versus non-obese patients, gene set enrichment analysis (GSEA) was performed for patients with overlapping gene expression data (FIGO1 grade 1-2 with BMI 20-30: n=67, BMI>30: n=43). Gene sets related to estrogen response, *MYC*-target gene sets, inflammation and hypoxia were significantly enriched in patients with BMI>30 (Supplementary Table 3). For the group with BMI 20-30, false discovery rate (FDR)-values were not sufficiently strong to draw relevant conclusions.

### Non-obese patients with FIGO stage 1, ERα positive tumors have better survival when PI3K pathway is activated

**Figure 3:**
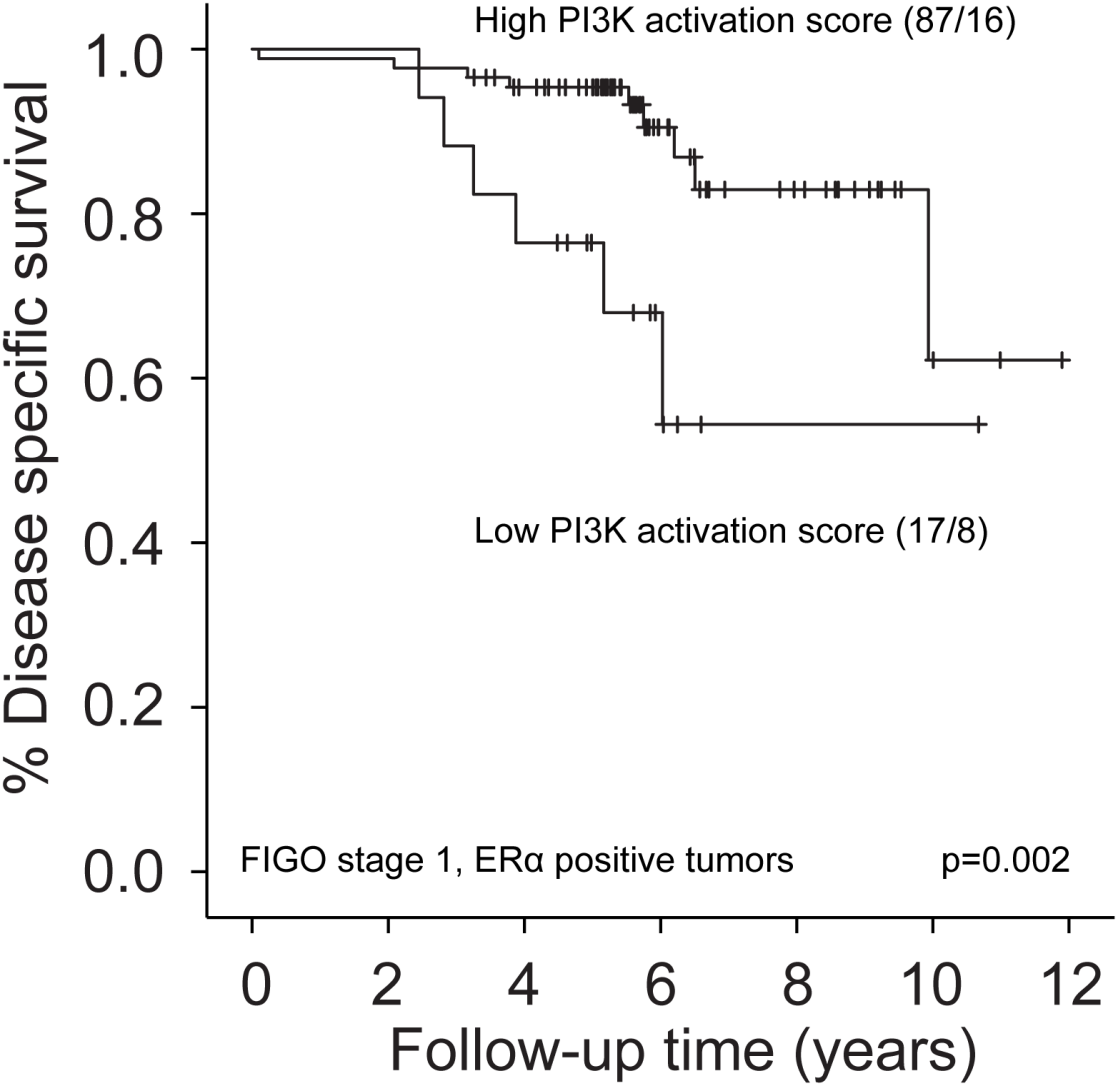
Survival according to PI3K-activation in non-obese patients with FIGO stage 1, ERα positive tumors Low PI3K activation score by RPPA (lowest quartile) was associated with reduced disease specific survival compared to high PI3K activation score (upper three quartiles).

We further assessed the effect of PI3K-activation in non-obese patients with FIGO stage 1, grade 1-2 tumors, and similarly in FIGO stage 1, ERα positive tumors, as almost all cancer-related deaths in these subgroups were observed in patients with BMI 20-30. No association was found between PI3K-activation and survival in FIGO stage 1, grade 1-2 tumors in the training set (p=0.22, data not shown). In contrast, in FIGO stage 1, ERα positive tumors, patients with low PI3K score had significantly reduced DSS compared to patients with higher PI3K scores (Figure 3). This could not be confirmed in the test sets (too low number of events in the Norwegian test set (n=1) and ERα status by IHC not available in MDACC test set). LIMMA was performed to assess differentially expressed proteins between patients with high and low PI3K score in this subgroup (training set). As expected, we found increased expression of PI3K pathway members, but also mTOR and MAPK-pathway members in tumors with high PI3K-activation. Tumors with low PI3K activity showed enrichment of apoptosis pathway and a signature of downstream hormone receptor signaling (Supplementary Table 4).

## DISCUSSION

In this descriptive and explorative study, we aimed to identify global protein expression patterns in relation to BMI in endometrioid endometrial carcinomas, using RPPA data from three independent cohorts. By applying different statistical methods and analytical approaches, two main trends were seen: increased MAPK pathway activity in non-obese patients, and increased hormone receptor signaling in obese patients. This was also observed in subgroup analyses of patients with FIGO stage 1 tumors, where survival was shown to vary according to BMI with significantly decreased survival for non-obese patients.

We found increasing BMI to correlate with increasing PR levels in the training cohort, also validated in the two test cohorts. These results are in line with a previous, partly overlapping, study including all histological subtypes, in which PR expression was assessed by IHC and mRNA arrays [15]. PR level assessed by RPPA was significantly and positively correlated with BMI in the study by Westin *et al.* [17], and our study now confirms this correlation in two new RPPA cohorts. The reasons for increased PR expression with increasing BMI are currently not well understood, but may relate to increasing estradiol levels with increasing BMI. In endometrial cancer cell lines, as estradiol has been shown to induce *PGR* mRNA expression [23], this may possibly lead to increased PR expression.

In the two Norwegian cohorts, BMI was correlated with p-ERα (S118) levels, also seen in subgroup analyses of patients with FIGO stage 1, grade 1-2 tumors (training cohort). In contrast, total ERα by RPPA, ERα assessed by IHC and *ESR1* mRNA were not correlated with BMI. Phosphorylation of ERα is thought to be important for receptor function, although relatively little is known about the relevance of the specific ERα phosphorylation sites *in vivo* [24]. ERα S118 phosphorylation can be induced by several mechanisms: directly by estradiol binding to ERα leading to phosphorylation by cyclin-dependent kinases, and indirectly by growth factors activating RTKs, leading to MAPK-activation and thereby ERα phosphorylation (ligand-independent activation) [24, 25]. However, MAPK pathway activation was negatively correlated with p-ERα (S118) levels and BMI in our data (Table 2). Thus, our data indicate that the direct route might be the main determinant of ERα S118 phosphorylation in obese patients, supported by a study from breast cancer indicating that estrogen-induced ERα S118 phosphorylation occurs independently of Erk1 and Erk2 (MAPK) [26].

Given the explanatory models wherein obese individuals have higher circulating levels of mediators including insulin, glucose, leptin and estrogen [7, 27–29], the negative correlations between BMI and MAPK- and RTK-signaling were somewhat unexpected. However, *in vitro* studies examining endometrial cancer cell lines have shown that glucose treatment induced Erk1 and Erk2 phosphorylation (p-MAPK) in short-term incubation, but that phosphorylation levels were reduced after long-term stimulation [30]. Our data underline the concept that obesity-mediated signaling happens through a complex network of pathways, likely influenced by a range of different mediators. Unfortunately, we currently do not have data available to examine metabolic signaling in these patients, which will be interesting to explore in future analyses. This also underscores that BMI alone is not a sufficient measure to capture the complexities of obesity at the individual patient level.

The observation that non-obese patients with FIGO stage 1, grade 1-2 tumors had reduced survival compared to obese patients is clinically relevant. The observed RTK pathway activation and downstream targets in the MAPK pathway in non-obese individuals requires further validation in larger, population based data sets. If confirmed, several novel targets for therapy could potentially be explored in this patient group. A recent window-of-opportunity trial with metformin, an oral biguanide used in the treatment of diabetes mellitus, showed reduction in p-MAPK expression in endometrial cancer patients [31], and could thus represent one promising agent for these patients.

GSEA revealed enrichment of gene sets related to estrogen response in the obese patients with FIGO stage 1, grade 1-2 tumors, in line with the observation of increased expression of activated p-ERα (S118) in this group. These tumors also showed enrichment of gene sets related to hypoxia, inflammation and immune response. Previous studies have linked a high level of epithelial infiltration of cytotoxic T-lymphocytes to improved endometrial cancer prognosis [32, 33]. Although inflammation is thought to promote tumor growth and enhance malignant progression [34], there is also emerging evidence that inflammation and hypoxia may recruit anti-tumor effectors, such as polymorphonuclear neutrophils, in endometrial cancer, and their presence has been linked to improved survival in both mice and humans [35]. Our data thus support the notion that tumor inflammation and increased immunogenic signaling might be features associated with better outcome in low stage, low grade, endometrioid endometrial cancer.

The lack of correlation between the MAPK signature proteins (all phospho-proteins) and mRNA level (Table 3) support the notion that relevant targets are not necessarily differentially expressed on transcriptional level. Previous studies have shown great variability in correlation between mRNA and protein expression data, and particularly phospho-protein levels [21, 22]. This again underlines the importance of a cross-platform approach, and especially studies at protein level, to identify clinically relevant targets.

In our subgroup survival analyses of FIGO stage 1 tumors, most events were seen in the non-obese group. In light of this, the specifically reduced survival seen for non-obese patients with low PI3K pathway activation score is interesting. Previous studies, which also included non-endometrioid tumors, have linked indicators of PI3K-activation to a more aggressive endometrial cancer phenotype [36, 37]. Analogously, PTEN loss, and thereby PI3K-activation, was associated with a tendency to reduced survival for non-obese patients in the study by Westin *et al.*, where the authors suggested that the impact of PI3K-activation likely is dependent on obesity [17]. Our data indicate that for non-obese patients with early stage, well-differentiated tumors (intact ERα expression), PI3K-activation conveys better survival, supporting the contention that the effect of PI3K-activation is context-dependent. The tumors with high PI3K-activation were characterized by MAPK- and mTOR-activation, and reduced apoptosis. Although we found that non-obese patients with low grade and low stage tumors had reduced survival and higher MAPK-levels compared to the obese group; according to our analyses, there seems to be a particularly aggressive subgroup of tumors within the subset of non-obese patients, driven by other mechanisms than MAPK-activation. Further studies are needed to clarify the impact of this observation.

In this study, we used two independent RPPA test cohorts to validate our findings, however, results were only partly reproduced. There may be many explanations to this. The two Norwegian sets were similar regarding patient characteristics; however, the smaller sample size may have underpowered the test set to confirm findings. In addition, this data set had shorter follow-up time, and thus fewer events. The MDACC set showed significant differences in most baseline characteristics compared to the Norwegian data sets (Supplementary Table 1), which remained when comparing patients from Caucasian descent only (data not shown), therefore subsequent analyses were performed including all patients. The patients in the Norwegian cohorts are in over 95% from Scandinavian descent.

A weakness of our study is that several of our results were not significant after adjustment for multiple testing. However, similar protein expression patterns were seen using different approaches and statistical methods, including increased PR levels and reduced p-MAPK (T202 Y204) levels in relation to BMI. These findings are therefore considered robust, and with potential clinical relevance. Another weakness is that the two Norwegian data sets could not be merged, due to technical challenges related to batch effects between RPPA data sets which were run at different time points. Thus, we potentially loose power to detect important differences. On the other hand, with this study design, we can consider findings confirmed in the test sets robust.

Obesity is a complex phenotypic trait to study, and is thought to influence many different cellular pathways and mechanisms [7–10]. Although BMI may be a too crude measure to reveal all important obesity-related alterations, especially on the individual level, we identify in the present study significant differences in protein expression pattern according to BMI in endometrial cancer patients. The inverse correlation between BMI and MAPK signaling has to our knowledge not been described previously, and merits further investigation.

## MATERIALS AND METHODS

### Norwegian cohorts

Two independent cohorts of patients, which form part of the larger Momatec (Molecular Markers in Treatment of Endometrial Cancer) study [38], treated for endometrioid endometrial cancer (EEC) at Haukeland University Hospital, Bergen, Norway, between 2001 and 2015 were used in this study. The included patients gave written informed consent, and the study was approved by the local ethical committee (REK vest, IRB number 2009/2315). All patients included in the study underwent hysterectomy with bilateral salpingo-oophorectomy, and fresh frozen tumor tissue was collected during primary surgery. Tumors were surgically staged according to the International Federation of Gynecology and Obstetrics (FIGO) 2009 criteria [39]. Clinical data and histopathological characteristics were obtained by review of the medical records and by correspondence with the primary physicians and gynecologists responsible for follow-up controls. BMI was registered at the time of diagnosis, and calculated as weight divided by height squared (kg/m^2^). To eliminate any biological effects potentially related to severe cachexia, patients with a BMI<20 were not included. The primary training cohort consisted of 272 patients treated between 2001 and 2013. The test cohort included 68 patients treated between 2011 and 2015. Median follow-up time for survivors was 5.3 years for the training cohort (range 2.3 – 12.3), and 2.8 years (range 0.3 – 4.5) for the test cohort.

### MDACC cohort

An external test cohort consisting of 178 patients treated for EEC at the M.D. Anderson Cancer Center (MDACC), Houston, TX, USA, between 2000 and 2009, with available BMI measurements (BMI≥20) and RPPA data was also used. Median follow-up time for survivors was 7.1 years (range 0.1 – 14.7). This cohort differed from the Norwegian cohorts on multiple aspects (Supplementary Table 1).

### Reverse phase protein array (RPPA)

Reverse phase protein array analysis was performed according to previously described protocols [20, 40]. Briefly, protein lysates (1μg/mL) were prepared from fresh frozen endometrial tumor tissue, five-fold serial diluted and printed on nitrocellulose-coated slides by an Aushon 2470 arrayer (Aushon BioSystems, Billerica, MA, USA). The slides were probed with validated primary antibodies targeting total protein and phospho-proteins validated for RPPA use, followed by detection with appropriate Biotinylated secondary antibodies. The signals were visualized by streptavidin-conjugated HRP and DAB colorimetric reaction. Slides were scanned and quantitated using the ArrayPro Analyzer software (Meyer Instruments, Inc., Houston, TX, USA). Relative protein levels were determined using Supercurve (R package, available at http://r-forge.r-project.org/projects/supercurve/) [41]. Median centering across antibodies was applied to correct for protein loading [40]. For each of the data sets, all samples were printed on the same slide, thus analyzed in the same run. Because of batch effects between the slides (and thus data sets) produced in the different runs that could not be removed by normalization procedures, the three data sets were analyzed separately. There were 163 overlapping antibodies between the three data sets, used for further analysis (Supplementary Materials). Phospho-protein nomenclature follows this system: p-ERα (S118), meaning ERα protein phosphorylated on serine residue 118.

### Pathway signatures

Previously defined pathway predictors from the TCGA pan-cancer proteomic project were used to calculate pathway activation scores for 12 pathways as described previously [18]. A full list of pathway members included in calculation of each score is provided in Supplementary Materials. To explore pathway activation scores without predefined cut-off values according to survival, the scores were divided into quartiles. Groups with similar survival in Kaplan–Meier analysis were merged.

### Immunohistochemistry

Immunohistochemical staining for ERα, PR and AR on tissue microarray (TMA) sections was available for n=266/268/226 tumors respectively in the training cohort, and n=55/56/54 tumors in the Norwegian test cohort. Slides were stained and evaluated as previously described [42–44]. The TMA method has been described and validated previously [45, 46]. Briefly, a staining index (SI) was calculated based on the area of nuclear staining (graded 0-3) multiplied by the intensity of the staining (graded 0-3). For correlation analyses, staining indices were assessed as ordinal variables (range 0-9).

### Gene expression microarrays

Gene expression data were available for 180 patients overlapping with the Norwegian RPPA cohorts (n=128 training cohort, n=52 test cohort), and were used to cross-validate findings from RPPA on mRNA level. RNA was extracted from fresh frozen tumor tissue using RNeasy Mini Kit (Qiagen, Hilden, Germany), before hybridization to Agilent Whole Human Genome Microarray Kit, 44k (Catalogue number G4112F) according to the manufacturers’ instructions and as previously described [42]. Arrays were scanned using the Agilent microarray scanner bundle. The software J-express (www.molmine.com/jexpress) was used to analyze the data, with median spot applied as intensity measure. The data set was quantile normalized and log2 transformed before analysis. For genes represented by multiple probes, the probe with highest absolute correlation coefficient was selected when comparing RPPA and mRNA expression levels. Gene set enrichment analysis (GSEA) (www.broadinstitute.org/gsea) was performed on the expression data set using the maximum probe expression values (30,500 probes) [47]. Molecular signatures database (MSigDB, version 5.1) data sets Hallmark and c2 (curated gene sets) were used. FDR <1% was set as cut-off level when determining significantly enriched gene sets.

### Statistics

Associations between categorical variables were assessed by Pearson Chi-squared test. Correlations between continuous variables were tested using Spearman rank correlation (rho, ρ). Mann-Whitney test and Kruskal-Wallis test were applied to assess differential distribution of continuous variables between two or more than two groups, respectively. Univariable survival analyses were performed by the Kaplan–Meier method, assessing survival differences between groups by the log-rank test (Mantel-Cox). When multiple ordered categories were included, linear trend test for factor levels was used. Multivariable survival analyses were performed using the Cox proportional hazards regression model. For analyses of DSS, patients were followed from the date of surgery, and death from endometrial cancer was registered as event. Patients who died from other causes were censored at the date of death. The MDACC test set did not report data on DSS, instead PFS was used. A linear model for microarray analysis (LIMMA) implemented in Bioconductor was applied to assess differentially expressed proteins between two groups [48]. To define a linear regression model identifying the proteins that best predicted BMI groups, Akaike Information Criterion (AIC) stepwise selection modelling was performed [49]. A score was then calculated based on the intercept and weighting for each variable in the equation. For the creation of this model, BMI was dichotomized in two groups (BMI 20-30 and BMI ≥35), excluding the group with BMI 30-35 to increase detection of significant differences. Receiver operating characteristic (ROC) curves were generated to evaluate the score’s ability to predict BMI groups. Analyses were performed using R version 3.3.2 (https://cran.r-project.org/) and Statistical Package for the Social Sciences (SPSS) version 23 (IBM SPSS Statistics, Armonk, NY, USA).

## SUPPLEMENTARY MATERIALS FIGURES AND TABLES





## Abbreviations

AIC: Akaike information criterion
AMPK: AMPK-activated protein kinase
AR: Androgen receptor
AUC: Area under the curve
BMI: Body mass index
DAB: Diaminobenzidine
DSS: Disease specific survival
EC: Endometrial cancer
EEC: Endometrioid endometrial cancer
ERα: Estrogen receptor alpha
*ESR1*: Estrogen receptor 1
FDR: False discovery rate
FIGO: International Federation of Gynecology and Obstetrics
GSEA: Gene set enrichment analysis
HRP: Horseradish peroxidase
IHC: Immunohistochemistry
LIMMA: Linear model for microarray analysis
MAPK: Mitogen-activated protein kinase
MDACC: M.D. Anderson cancer center
PFS: Progression free survival
PI3K: Phosphatidylinositide 3-kinase
*PGR*: Progesterone receptor gene
PR: Progesterone receptor
PTEN: Phosphatase and tensin homolog
ROC: Receiver operating characteristic
RPPA: Reverse phase protein array
RTK: Receptor tyrosine kinase
SI: Staining index
TCGA: The Cancer Genome Atlas
TMA: Tissue microarray
WHO: World Health Organization
