# Supplementary material for: Proteomic profiling of endometrioid endometrial cancer reveals differential expression of hormone receptors and MAPK signaling proteins in obese versus non-obese patients
Source: Oncotarget. 2017 Oct 31;8(63):106989–7001. doi: 10.18632/oncotarget.22203 (PMC5739790; doi:10.18632/oncotarget.22203)
Supplement: Supplementary file 1 [file oncotarget-08-106989-s001.pdf]

Proteomic profiling of endometrioid endometrial cancer reveals differential expression of hormone receptors and MAPK signaling proteins in obese versus non-obese patients

SUPPLEMENTARY MATERIALS

A. Overview of 163 proteins and phospho-proteins employed in the RPPA-assay overlapping between the three data sets used in this study, with antibody information

See Supplementary File 1

B. Overview of proteins and phospho-proteins used for calculation of pathway activation scores (Akbani *et al*, Nature Commun, 2014). Pathway members colored to aid visualisation

See Supplementary File 1

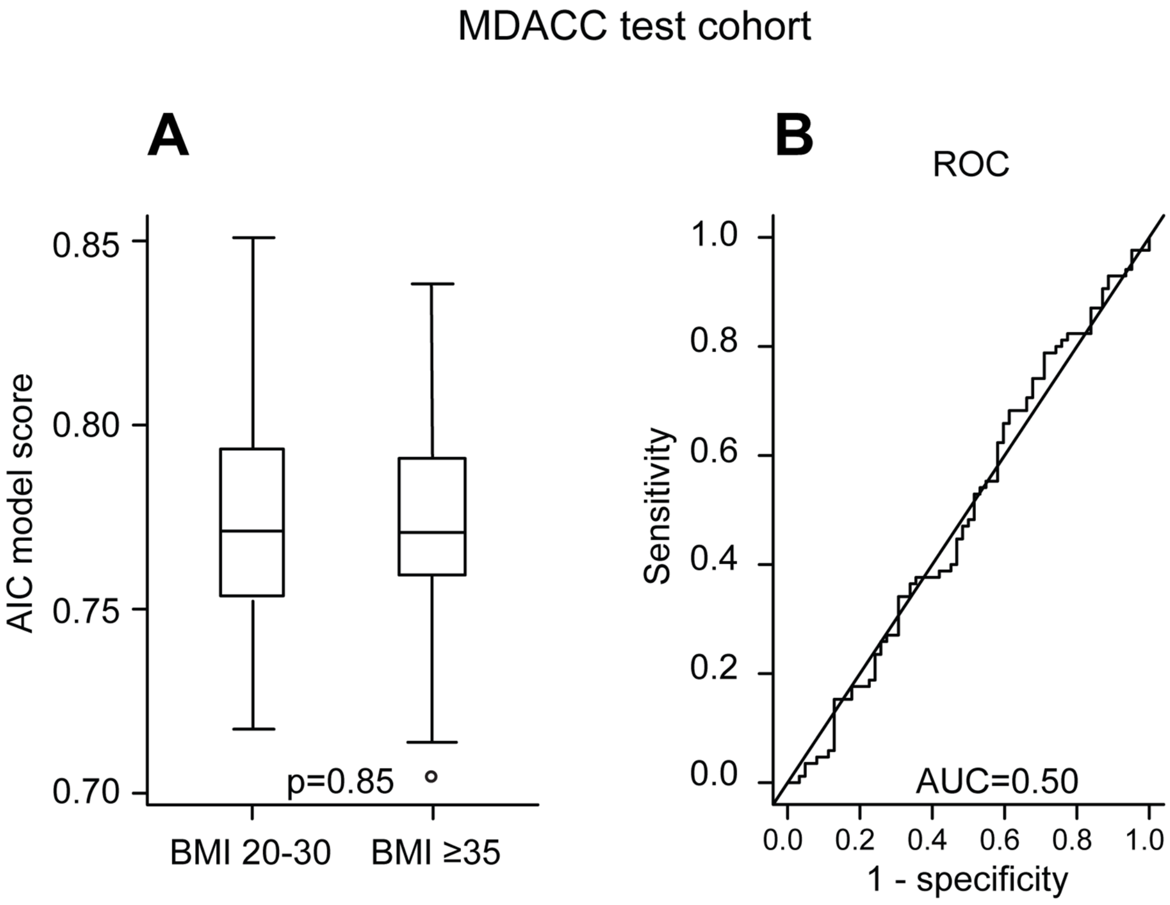

Supplementary Figure 1: Distribution of model score from 5 proteins in MDACC data set according to BMI groups as defined in Figure 1. **(A)** No difference in AIC model score distribution according to BMI groups. **(B)** ROC-curve for prediction models in MDACC test set.

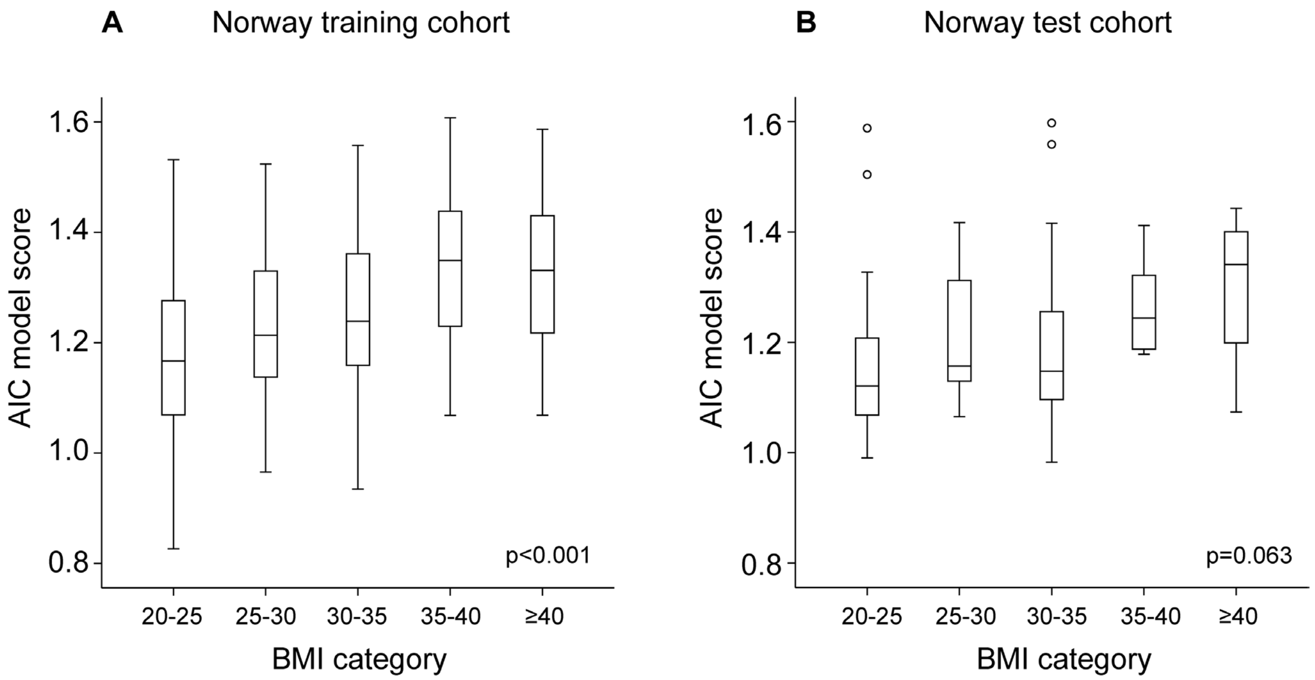

Supplementary Figure 2: Display of AIC-derived model score in BMI classes as defined by the WHO criteria. Distribution of BMI associated protein signature in WHO BMI categories for **(A)**. Norway training set and **(B)**. Norway test set. P-value: Kruskal-Wallis test.

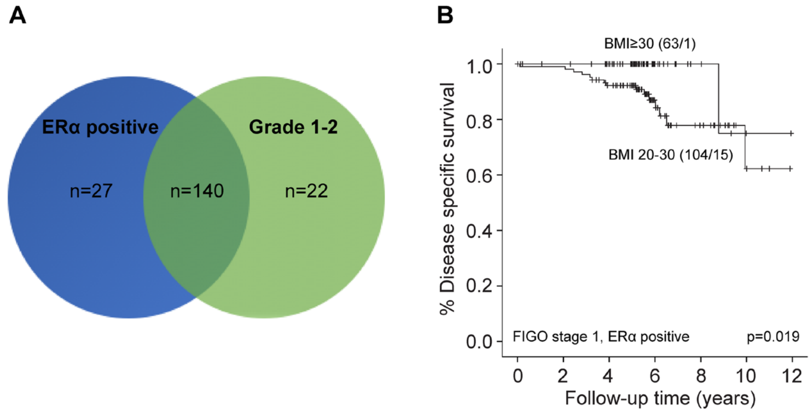

C

**Protein/*pathway***	**LogFC high vs low BMI**	**P-value (unadjusted)**
p-ER (S118)*	0.32	0.002
p-MAPK (T202 Y204)*	-0.36	0.010
p-JNK (T183 Y185)*	-0.13	0.012
PR	0.15	0.014
Hormone_a	1.00	0.018
ETS1*	-0.15	0.026
YAP*	0.09	0.032
p-MEK1 (S217 S221)*	-0.12	0.033
p-PEA15 (S116)	-0.23	0.036
p-SRC (Y527)	-0.22	0.040

Supplementary Figure 3: Patients with FIGO stage 1, ERα positive tumors have similar survival and protein expression pattern as patients with FIGO stage 1, grade 1-2 tumors according to BMI groups. **(A)** Venn diagram showing overlap between patients with FIGO stage 1, grade 1-2 tumors and FIGO stage 1, ERα positive tumors (assessed by immunohistochemistry). **(B)** Similar pattern of disease specific survival for obese versus non-obese patients with FIGO stage 1, ERα positive tumors (compare to Figure 2B). **(C)** Differentially expressed proteins by LIMMA in tumors from obese versus non-obese patients. * indicates the same protein was differentially expressed in FIGO stage 1, grade 1-2 tumors (compare to Supplementary Table 2).

Abbreviations: LogFC: Log2 fold change.

Supplementary Table 1: Comparison between Norwegian training cohort and MDACC validation cohort

	Norway training cohort (n=272) 2001 – 2013	MDACC test cohort (n=178) 2001 – 2009	
	n (%)	n (%)	p-value[1]
**FIGO stage**			0.02
I	204 (75)	110 (62)	
II	24 (9)	25 (14)	
III	34 (13)	29 (16)	
IV	10 (4)	13 (7)	
**Grade**[2]			0.03
Grade 1-2	203 (76)	145 (84)	
Grade 3	65 (24)	27 (16)	
**Age (mean, SD)**	65.2 (11.5)	60.5 (12.9)	<0.001
**BMI (mean, SD)**	29.4 (6.9)	36.3 (11.2)	<0.001
**BMI groups**			<0.001
20-25	84 (31)	21 (12)	
25-30	84 (31)	41 (23)	
30-35	55 (20)	30 (17)	
35-40	27 (10)	25 (14)	
≥40	22 (8)	61 (34)	

Abbreviations: BMI: Body mass index; FIGO: International Federation of Gynecology and Obstetrics; MDACC: M.D. Anderson Cancer Center; SD: Standard deviation. n=number of patients.

[1] Pearson Chi-square test for categorical variables, t-test for continuous variables.

[2] Grade missing for 4 tumors in Norway training cohort, 6 tumors in MDACC test cohort.

Supplementary Table 2: Differentially expressed proteins and pathways in tumors from obese (BMI≥30) versus non-obese (BMI20-30) patients with FIGO stage 1, grade 1-2 disease (training set, n=162)

**Protein/*pathway***	**Log2 Fold change**	**p-value (unadjusted)**
p-MAPK (T202 Y204)	-0.40	0.004
p-ERα (S118)	0.29	0.007
p-JNK (T183 Y185)	-0.13	0.008
YAP	0.10	0.013
p-MEK1 (S217 S221)	-0.13	0.025
P38	-0.20	0.034
RTK	-0.67	0.042
ETS1	-0.14	0.050

Abbreviations: BMI: Body mass index; FIGO: International Federation of Gynecology and Obstetrics. p- indicates phospho-protein (site of phosphorylation in parenthesis).

Supplementary Table 3: Gene set enrichment analysis (GSEA) comparing FIGO stage 1, grade 1-2 tumors arising in obese (BMI≥30, n=43) versus non-obese (BMI 20-30, n=67) patients using Hallmark and c5 (curated) gene sets

Rank	Gene set	Size	p-value	FDR (%)
Hallmark gene sets				
1	HALLMARK_MYC_TARGETS_V2	50	0.0	0.0
2	HALLMARK_MYC_TARGETS_V1	178	0.0	0.0
3	HALLMARK_TNFA_SIGNALING_VIA_NFKB	188	0.0	0.0
5	HALLMARK_ESTROGEN_RESPONSE_EARLY	186	0.0	0.04
6	HALLMARK_INTERFERON_GAMMA_RESPONSE	189	0.0	0.03
8	HALLMARK_HYPOXIA	189	0.0	0.08
9	HALLMARK_ALLOGRAFT_REJECTION	193	0.0	0.07
10	HALLMARK_INTERFERON_ALPHA_RESPONSE	90	0.0	0.08
11	HALLMARK_INFLAMMATORY_RESPONSE	194	0.0	0.46
14	HALLMARK_IL6_JAK_STAT3_SIGNALING	86	0.01	0.72
15	HALLMARK_ESTROGEN_RESPONSE_LATE	196	0.0	0.83
Curated gene sets				
1	SCHUHMACHER_MYC_TARGETS_UP	75	0.0	0.0
3	WINTER_HYPOXIA_UP	81	0.0	0.0
4	SANA_TNF_SIGNALING_UP	78	0.0	0.0
5	HINATA_NFKB_TARGETS_FIBROBLAST_UP	83	0.0	0.0
6	MANALO_HYPOXIA_DN	256	0.0	0.0
7	DUTERTRE_ESTRADIOL_RESPONSE_6HR_UP	210	0.0	0.0
11	SCHLOSSER_MYC_TARGETS_AND_SERUM_RESPONSE_DN	46	0.0	0.07
12	SCHLOSSER_MYC_TARGETS_AND_SERUM_RESPONSE_UP	45	0.0	0.06
13	JAIN_NFKB_SIGNALING	70	0.0	0.07
17	THEILGAARD_NEUTROPHIL_AT_SKIN_WOUND_UP	73	0.0	0.11
18	KARLSSON_TGFB1_TARGETS_UP	111	0.0	0.11
20	PID_MYC_ACTIV_PATHWAY	76	0.0	0.15
23	MENSE_HYPOXIA_UP	79	0.0	0.15
26	PHONG_TNF_TARGETS_UP	61	0.0	0.19
27	WINTER_HYPOXIA_METAGENE	227	0.0	0.2
28	DANG_REGULATED_BY_MYC_UP	66	0.0	0.29
30	MASSARWEH_RESPONSE_TO_ESTRADIOL	54	0.0	0.3
31	HINATA_NFKB_TARGETS_KERATINOCYTE_UP	91	0.0	0.3
34	GALINDO_IMMUNE_RESPONSE_TO_ENTEROTOXIN	80	0.0	0.38
37	REACTOME_TAK1_ACTIVATES_NFKB_BY_PHOSPHORYLATION_AND_ACTIVATION_OF_IKKS_COMPLEX	20	0.0	0.48
40	BILD_MYC_ONCOGENIC_SIGNATURE	170	0.0	0.64
41	DER_IFN_BETA_RESPONSE_UP	96	0.0	0.64
42	GROSS_HYPOXIA_VIA_HIF1A_UP	68	0.0	0.67
43	ZHANG_RESPONSE_TO_IKK_INHIBITOR_AND_TNF_UP	199	0.0	0.65
47	WACKER_HYPOXIA_TARGETS_OF_VHL	13	0.0	0.85
49	SEKI_INFLAMMATORY_RESPONSE_LPS_UP	74	0.0	0.88
50	DUTERTRE_ESTRADIOL_RESPONSE_24HR_UP	294	0.0	0.88
52	COLLER_MYC_TARGETS_UP	23	0.0	0.93
53	MENSSEN_MYC_TARGETS	49	0.0	0.93
54	SCHLOSSER_MYC_TARGETS_REPRESSED_BY_SERUM	140	0.0	0.96

Selected gene sets enriched in obese patients with FDR < 1% are displayed.
Abbreviations: BMI: Body mass index; FDR: False discovery rate; FIGO: International Federation of Gynecology and Obstetrics. n=number of patients. Size indicates number of genes in reference gene set (available at www.msigdb.org).
Colors indicate category of the gene set: green: Myc-related gene set; yellow: inflammation/immune activation related gene set; red: estrogen related gene set; blue: hypoxia related gene set.

Supplementary Table 4: Top ranked proteins/pathways differentially expressed between high and low PI3K score in non-obese patients with FIGO stage 1, ERα positive tumors (immunohistochemistry)

A. Norway training cohort (n=104)

**Protein/*pathway***	**Log2 fold change (high versus low PI3K score)**	**FDR adjusted p-value**
PI3K_AKT	5.8	4.10e-26
p-AKT (S473)	1.9	1.14e-19
p-AKT (T308)	1.6	1.62e-19
p-GSK3AB(S21 S9)	1.1	1.15e-17
p-P38 (T180 Y182)	1.0	9.11e-12
p-SRC (Y527)	0.8	3.85e-10
p-RB (S807 S811)	0.8	1.84e-09
TSC_mTOR	2.6	2.21e-09
G6PD	-0.6	2.86e-08
RAS_MAPK	4.5	7.76e-08
p-PKCPANBII (S660)	0.5	2.00e-07
p-NDRG1 (T346)	0.7	3.91e-06
p-NFKBP65 (S536)	0.7	7.39e-06
p-MAPK (T202 Y204)	0.7	1.35e-05
Hormone_b	-1.5	4.78e-05
Apoptosis	-2.1	7.12e-05
PDCD4	0.6	0.0002
CASPASE7CLEAVED (D198)	-0.8	0.0004

B. Norway test cohort (n=20)

**Protein/*pathway***	**Log2 fold change (high versus low PI3K score)**	**p-value (unadjusted)**
PI3K_AKT	4.4	4.32e-07
p-AKT (T308)	0.6	0.0025
Hormone_b	-1.4	0.0091
MEK1	-0.8	0.0133
PDCD4	0.6	0.0261
p-AKT (S473)	0.7	0.0481

Proteins/pathways with fold change ±0.5 or larger are displayed.
Abbreviations: ERα: Estrogen receptor alpha; FDR: False discovery rate; FIGO: International Federation of Gynecology and Obstetrics; PI3K: Phosphatidylinositide 3-kinase. p- indicates phospho-protein (site of phosphorylation in parenthesis).
