# Supplementary material for: Proteomic profiling of endometrioid endometrial cancer reveals differential expression of hormone receptors and MAPK signaling proteins in obese versus non-obese patients
Source: Oncotarget. 2017 Oct 31;8(63):106989–7001. doi: 10.18632/oncotarget.22203 (PMC5739790; doi:10.18632/oncotarget.22203)
Supplement: Supplementary file 2 [file oncotarget-08-106989-s002.docx]

**Supplementary Materials**

1. **Overview of RPPA target proteins**
2. **Proteins included in pathway score analysis**

**A. Overview of 163 proteins and phospho-proteins employed in the RPPA-assay overlapping between the three data sets used in this study, with antibody information**

| **Official Ab Name** | **Ab Name Reported** | **Gene Name** | **Company** | **Catalog #** | **Species** | **RPPA Dilution** |  |
| --- | --- | --- | --- | --- | --- | --- | --- |
| 14-3-3 beta | 1433BETA | YWHAB | Santa Cruz | sc-628 | Rabbit | 1:75 |  |
| 14-3-3 epsilon | 1433EPSILON | YWHAE | Santa Cruz | sc-23957 | Mouse | 1:50 |  |
| 14-3-3 zeta | 1433ZETA | YWHAZ | Santa Cruz | sc-1019 | Rabbit | 1:5000 |  |
| 4E-BP1 | 4EBP1 | EIF4EBP1 | CST | 9452 | Rabbit | 1:100 |  |
| 4E-BP1 (phospho S65) | 4EBP1PS65 | EIF4EBP1 | CST | 9456 | Rabbit | 1:250 |  |
| 53BP1 | 53BP1 | TP53BP1 | CST | 4937 | Rabbit | 1:300 |  |
| Acetyl CoA Carboxylase (phospho S79) | ACCPS79 | ACACA, ACACB | CST | 3661 | Rabbit | 1:500 |  |
| Acetyl CoA Carboxylase 1 | ACC1 | ACACA | Abcam | ab45174 | Rabbit | 1:20000 |  |
| ADAR1 | ADAR1 | ADAR | Abcam | ab88574 | Mouse | 1:200 |  |
| Akt | AKT | AKT1,2,3 | CST | 4691 | Rabbit | 1:10000 |  |
| Akt (phospho S473) | AKTPS473 | AKT1,2,3 | CST | 9271 | Rabbit | 1:150 |  |
| Akt (phospho T308) | AKTPT308 | AKT1,2,3 | CST | 2965 | Rabbit | 1:500 |  |
| AMPK alpha | AMPKA | PRKAA1 | CST | 2532 | Rabbit | 1:200 |  |
| AMPK alpha (phospho T172) | AMPKAPT172 | PRKAA1 | CST | 2535 | Rabbit | 1:100 |  |
| Androgen Receptor | AR | AR | Abcam | ab52615 | Rabbit | 1:100 |  |
| Annexin I | ANNEXIN1 | ANXA1 | BD Biosciences | 610066 | Mouse | 1:5000 |  |
| Annexin VII | ANNEXINVII | ANXA7 | BD Biosciences | 610668 | Mouse | 1:30 |  |
| ATM | ATM | ATM | CST | 2873 | Rabbit | 1:250 |  |
| Bad (phospho S112) | BADPS112 | BAD | CST | 9291 | Rabbit | 1:50 |  |
| Bak | BAK | BAK1 | Abcam | ab32371 | Rabbit | 1:30 |  |
| BAP1 | BAP1C4 | BAP1 | Santa Cruz | sc-28383 | Mouse | 1:125 |  |
| Bax | BAX | BAX | CST | 2772 | Rabbit | 1:100 |  |
| Bcl2 | BCL2 | BCL2 | Dako | M0887 | Mouse | 1:50 |  |
| Bcl-xL | BCLXL | BCL2L1 | CST | 2762 | Rabbit | 1:100 |  |
| Beclin | BECLIN | BECN1 | Santa Cruz | sc-10086 | Goat | 1:250 |  |
| beta Catenin | BCATENIN | CTNNB1 | CST | 9562 | Rabbit | 1:1500 |  |
| Bid | BID | BID | Abcam | ab32060 | Rabbit | 1:30 |  |
| Bim | BIM | BCL2L11 | Abcam | ab32158 | Rabbit | 1:400 |  |
| B-Raf | BRAF | BRAF | Abcam | ab33899 | Rabbit | 1:75 |  |
| B-Raf (phospho S445) | BRAFPS445 | BRAF | CST | 2696 | Rabbit | 1:1000 |  |
| c-IAP2 | CIAP | BIRC3 | CST | 3130 | Rabbit | 1:750 |  |
| Caspase-7 (cleaved D198) | CASPASE7CLEAVEDD198 | CASP7 | CST | 9491 | Rabbit | 1:75 |  |
| Caveolin-1 | CAVEOLIN1 | CAV1 | CST | 3238 | Rabbit | 1:5000 |  |
| CD31 | CD31 | PECAM1 | Dako | M0823 | Mouse | 1:30 |  |
| CD49b | CD49B | ITGA2 | BD Biosciences | 611016 | Mouse | 1:50 |  |
| CDK1 | CDK1 | CDK1 | Abcam | ab32384 | Rabbit | 1:1000 |  |
| Chk1 | CHK1 | CHEK1 | CST | 2360 | Mouse | 1:250 |  |
| Chk2 | CHK2 | CHEK2 | CST | 3440 | Mouse | 1:50 |  |
| Chk2 (phospho T68) | CHK2PT68 | CHEK2 | CST | 2197 | Rabbit | 1:125 |  |
| c-Jun ( phospho S73) | CJUNPS73 | JUN | CST | 9164 | Rabbit | 1:30 |  |
| c-Kit | CKIT | KIT | Abcam | ab32363 | Rabbit | 1:30 |  |
| Claudin 7 | CLAUDIN7 | CLDN7 | Novus Biologicals | NB100-91714 | Rabbit | 1:300 |  |
| c-Met (phospho Y1234/Y1235) | CMETPY1235 | MET | CST | 3129 | Rabbit | 1:100 |  |
| c-Myc | CMYC | MYC | Santa Cruz | sc-764 | Rabbit | 1:125 |  |
| COL6A1 | COLLAGENVI | COL6A1 | Santa Cruz | sc-20649 | Rabbit | 1:5000 |  |
| C-Raf (phospho S338) | CRAFPS338 | RAF1 | CST | 9427 | Rabbit | 1:100 |  |
| C-Raf/Raf-1 | CRAF | RAF1 | Millipore | 04-739 | Rabbit | 1:200 |  |
| Cyclin B1 | CYCLINB1 | CCNB1 | Epitomics | 1495-1 | Rabbit | 1:1500 |  |
| Cyclin D1 | CYCLIND1 | CCND1 | Santa Cruz | sc-718 | Rabbit | 1:200 |  |
| Cyclin E1 | CYCLINE1 | CCNE1 | Santa Cruz | sc-247 | Mouse | 1:30 |  |
| E-Cadherin | ECADHERIN | CDH1 | CST | 3195 | Rabbit | 1:300 |  |
| eEF2 | EEF2 | EEF2 | CST | 2332 | Rabbit | 1:50 |  |
| eEF2K | EEF2K | EEF2K | CST | 3692 | Rabbit | 1:50 |  |
| EGFR | EGFR | EGFR | CST | 2232 | Rabbit | 1:100 |  |
| EGFR (phospho Y1173) | EGFRPY1173 | EGFR | Abcam | ab32578 | Rabbit | 1:50 |  |
| eIF4E | EIF4E | EIF4E | CST | 9742 | Rabbit | 1:75 |  |
| eIF4G | EIF4G | EIF4G1 | CST | 2498 | Rabbit | 1:1000 |  |
| ErbB2/HER2 | HER2 | ERBB2 | Lab Vision | MS-325-P1 | Mouse | 1:3000 |  |
| ErbB2/HER2 (phospho Y1248) | HER2PY1248 | ERBB2 | R&D Systems | AF1768 | Rabbit | 1:1500 |  |
| ErbB3/HER3 | HER3 | ERBB3 | Santa Cruz | sc-285 | Rabbit | 1:300 |  |
| ErbB3/HER3 (phospho Y1289) | HER3PY1289 | ERBB3 | CST | 4791 | Rabbit | 1:50 |  |
| ERRFI1/MIG6 | MIG6 | ERRFI1 | Sigma-Aldrich | WH0054206M1 | Mouse | 1:50 |  |
| Estrogen Receptor | ER | ESR1 | Lab Vision | RM-9101 | Rabbit | 1:40 |  |
| Estrogen Receptor alpha (Phospho S118) | ERPS118 | ESR1 | Abcam | ab32396 | Rabbit | 1:1000 |  |
| Ets-1 | ETS1 | ETS1 | Bethyl | A303-501A | Rabbit | 1:100 |  |
| Fatty Acid Synthase | FASN | FASN | CST | 3180 | Rabbit | 1:1000 |  |
| Fibronectin | FIBRONECTIN | FN1 | Epitomics | 1574-1 | Rabbit | 1:10000 |  |
| FoxM1 | FOXM1 | FOXM1 | CST | 5436 | Rabbit | 1:30 |  |
| FoxO3a | FOXO3A | FOXO3 | CST | 2497 | Rabbit | 1:25 |  |
| FoxO3a (phospho S318/S321) | FOXO3APS318S321 | FOXO3 | CST | 9465 | Rabbit | 1:30 |  |
| G6PD | G6PD | G6PD | CST | 8866 | Rabbit | 1:1000 |  |
| Gab2 | GAB2 | GAB2 | CST | 3239 | Rabbit | 1:300 |  |
| GAPDH | GAPDH | GAPDH | Life Technologies | AM4300 | Mouse | 1:50000 |  |
| GATA3 | GATA3 | GATA3 | BD Biosciences | 558686 | Mouse | 1:300 |  |
| GSK-3alpha/beta | GSK3AB | GSK3A, GSK3B | Santa Cruz | sc-7291 | Mouse | 1:750 |  |
| GSK-3alpha/beta (phospho S21/S9) | GSK3ABPS21S9 | GSK3A, GSK3B | CST | 9331 | Rabbit | 1:200 |  |
| Heregulin | HEREGULIN | NRG1 | CST | 2573 | Rabbit | 1:30 |  |
| HSP27 (phospho S82) | HSP70 | HSBP1 | CST | 2401 | Rabbit | 1:75 |  |
| IGFBP2 | IGFBP2 | IGFBP2 | CST | 3922 | Rabbit | 1:50 |  |
| INPP4b | INPP4B | INPP4B | CST | 4039 | Rabbit | 1:25 |  |
| IRS1 | IRS1 | IRS1 | Millipore | 06-248 | Rabbit | 1:400 |  |
| Jak2 | JAK2 | JAK2 | CST | 3230 | Rabbit | 1:750 |  |
| JNK/SAPK (phospho T183/Y185) | JNKPT183Y185 | MAPK8 | CST | 4668 | Rabbit | 1:30 |  |
| JNK2 | JNK2 | MAPK9 | CST | 4672 | Rabbit | 1:30 |  |
| Lck | LCK | LCK | CST | 2752 | Rabbit | 1:100 |  |
| MAPK (phospho T202/Y204) | MAPKPT202Y204 | MAPK1, MAPK3 | CST | 4377 | Rabbit | 1:30 |  |
| MEK1 | MEK1 | MAP2K1 | Abcam | ab32576 | Rabbit | 1:1500 |  |
| MEK1 (phospho S217/S221) | MEK1PS217S221 | MAP2K1 MAP2K2 | CST | 9154 | Rabbit | 1:50 |  |
| Merlin/NF2 | NF2 | NF2 | Novus Biologicals | 22710002 | Rabbit | 1:250 |  |
| MSH6 | MSH6 | MSH6 | Novus Biologicals | 22030002 | Rabbit | 1:1000 |  |
| mTOR | MTOR | MTOR | CST | 2983 | Rabbit | 1:1000 |  |
| mTOR (phospho S2448) | MTORPS2448 | MTOR | CST | 2971 | Rabbit | 1:50 |  |
| Myosin IIa (phospho S1943) | MYOSINIIAPS1943 | MYH9 | CST | 5026 | Rabbit | 1:1000 |  |
| N-Cadherin | NCADHERIN | CDH2 | CST | 4061 | Rabbit | 1:30 |  |
| NDRG1 (phospho T346) | NDRG1PT346 | NDRG1 | CST | 3217 | Rabbit | 1:100 |  |
| NF-kappaB p65 (phospho S536) | NFKBP65PS536 | RELA | CST | 3033 | Rabbit | 1:30 |  |
| Notch1 | NOTCH1 | NOTCH1 | CST | 3268 | Rabbit | 1:30 |  |
| N-Ras | NRAS | NRAS | Santa Cruz | sc-31 | Mouse | 1:50 |  |
| p21 | P21 | CDKN1A | Santa Cruz | sc-397 | Rabbit | 1:150 |  |
| p27 KIP 1 | P27 | CDKN1B | Abcam | ab32034 | Rabbit | 1:50 |  |
| p27/KIP 1 (phospho T198) | P27PT198 | CDKN1B | Abcam | ab64949 | Rabbit | 1:30 |  |
| p38 MAPK | P38MAPK | MAPK14 | CST | 9212 | Rabbit | 1:1500 |  |
| p38 MAPK (phospho T180/Y182) | P38PT180Y182 | MAPK14 | CST | 9211 | Rabbit | 1:50 |  |
| p53 | P53 | TP53 | CST | 9282 | Rabbit | 1:2500 |  |
| p70/S6K1 | P70S6K1 | RPS6KB1 | Abcam | ab32529 | Rabbit | 1:300 |  |
| PAI-1 | PAI1 | SERPINE1 | BD Biosciences | 612024 | Mouse | 1:100 |  |
| PARK7/DJ1 | DJ1 | PARK7 | Abcam | ab76008 | Rabbit | 1:5000 |  |
| Paxillin | PAXILLIN | PXN | Epitomics | 1500-1 | Rabbit | 1:500 |  |
| P-Cadherin | PCADHERIN | CDH3 | CST | 2130 | Rabbit | 1:50 |  |
| PCNA | PCNA | PCNA | CST | 2586 | Mouse | 1:1000 |  |
| Pdcd4 | PDCD4 | PDCD4 | Rockland | 600-401-965 | Rabbit | 1:750 |  |
| PDK1 | PDK1 | PDPK1 | CST | 3062 | Rabbit | 1:50 |  |
| PDK1 (phospho S241) | PDK1PS241 | PDPK1 | CST | 3061 | Rabbit | 1:50 |  |
| PEA-15 | PEA15 | PEA15 | CST | 2780 | Rabbit | 1:100 |  |
| PED/PEA-15 (phospho S116) | PEA15PS116 | PEA15 | Invitrogen | 44-836G | Rabbit | 1:1000 |  |
| PI3 Kinase p110 alpha | PI3KP110A | PIK3CA | CST | 4255 | Rabbit | 1:75 |  |
| PI3K p85 | PI3KP85 | PIK3R1 | Millipore | 06-195 | Rabbit | 1:15000 |  |
| PKCalpha | PKCAPS657 | PRKCA | CST | 2056 | Rabbit | 1:200 |  |
| PKC beta II (phospho S660) | PKCPANBIIPS660 | PRKCA, PRKCB PRKCD, PRKCE PRKCH, PRKCQ | CST | 9371 | Rabbit | 1:200 |  |
| PKC delta (phospho S664) | PKCDELTAPS664 | PRKCD | Millipore | 07-875 | Rabbit | 1:100 |  |
| PRAS40 (phospho T246) | PRAS40PT246 | AKT1S1 | Life Technologies | 441100G | Rabbit | 1:500 |  |
| PREX1 | PREX1 | PREX1 | Abcam | ab102739 | Rabbit | 1:150 |  |
| Progesterone Repector | PR | PGR | Abcam | ab32085 | Rabbit | 1:50 |  |
| PTEN | PTEN | PTEN | CST | 9552 | Rabbit | 1:500 |  |
| Rab11 | RAB11 | RAB11A,B | CST | 3539 | Rabbit | 1:30 |  |
| Rab25 | RAB25 | RAB25 | CST | 4314 | Rabbit | 1:30 |  |
| Rad50 | RAD50 | RAD50 | Millipore | 05-525 | Mouse | 1:100 |  |
| Rad51 | RAD51 | RAD51 | CST | 8875 | Rabbit | 1:30 |  |
| Raptor | RAPTOR | RPTOR | CST | 2280 | Rabbit | 1:300 |  |
| Rb (phospho S807/S811) | RBPS807S811 | RB1 | CST | 9308 | Rabbit | 1:500 |  |
| RBM15 | RBM15 | RBM15 | Novus Biologicals | 21390002 | Rabbit | 1:5000 |  |
| Rictor | RICTOR | RICTOR | CST | 2114 | Rabbit | 1:100 |  |
| Rictor (phospho T1135) | RICTORPT1135 | RICTOR | CST | 3806 | Rabbit | 1:200 |  |
| S6 (phospho S235/S236) | S6PS235S236 | RPS6 | CST | 2211 | Rabbit | 1:2500 |  |
| S6 (phospho S240/S244) | S6PS240S244 | RPS6 | CST | 2215 | Rabbit | 1:1000 |  |
| S6 Ribosomal Protein | S6 | RPS6 | CST | 2317 | Mouse | 1:1000 |  |
| SCD | SCD1 | SCD | Santa Cruz | sc-58420 | Mouse | 1:30 |  |
| SF2/ASF | SF2 | SRSF1 | Invitrogen | 32-4500 | Mouse | 1:150 |  |
| Shc (phospho Y317) | SHCPY317 | SHC1 | CST | 2431 | Rabbit | 1:30 |  |
| SHP-2 (phospho Y542) | SHP2PY542 | PTPN11 | CST | 3751 | Rabbit | 1:75 |  |
| Smad1 | SMAD1 | SMAD1 | Abcam | ab33902 | Rabbit | 1:750 |  |
| Smad3 | SMAD3 | SMAD3 | Abcam | ab40854 | Rabbit | 1:150 |  |
| Smad4 | SMAD4 | SMAD4 | Santa Cruz | sc-7966 | Mouse | 1:30 |  |
| Src | SRC | SRC | Millipore | 05-184 | Mouse | 1:200 |  |
| Src (phospho Y527) | SRCPY527 | SRC, YES1, FYN FGR | CST | 2105 | Rabbit | 1:30 |  |
| Src Family (phospho Y416) | SRCPY416 | SRC, LYN, FYN LCK, YES1, HCK | CST | 2101 | Rabbit | 1:500 |  |
| Stat3 (phospho Y705) | STAT3PY705 | STAT3 | CST | 9131 | Rabbit | 1:30 |  |
| Stat5a | STAT5A | STAT5A | Abcam | ab32043 | Rabbit | 1:250 |  |
| Stathmin 1 | STATHMIN | STMN1 | Abcam | ab52630 | Rabbit | 1:75 |  |
| Syk | SYK | SYK | Santa Cruz | sc-1240 | Mouse | 1:3000 |  |
| TAZ | TAZ | WWTR1 | CST | 4883 | Rabbit | 1:300 |  |
| TIGAR | TIGAR | C12ORF5 | Abcam | ab137573 | Rabbit | 1:100 |  |
| Transferrin Receptor | TFRC | TFRC | Novus Biologicals | 22500002 | Rabbit | 1:15000 |  |
| Transglutaminase II | TRANSGLUTAMINASE | TGM2 | Lab Vision | MS-224-P1 | Mouse | 1:150 |  |
| TSC1/Hamartin | TSC1 | TSC1 | CST | 4906 | Rabbit | 1:200 |  |
| TSC2/Tuberin (phospho T1462) | TUBERINPT1462 | TSC2 | CST | 3617 | Rabbit | 1:30 |  |
| Tuberin | TUBERIN | TSC2 | Abcam | ab32554 | Rabbit | 1:2500 |  |
| VEGF Receptor 2 | VEGFR2 | KDR | CST | 2479 | Rabbit | 1:12000 |  |
| XBP1 | XBP1 | XBP1 | Santa Cruz | sc-32136 | Goat | 1:200 |  |
| XRCC1 | XRCC1 | XRCC1 | CST | 2735 | Rabbit | 1:30 |  |
| YAP | YAP | YAP1 | Santa Cruz | sc-15407 | Rabbit | 1:200 |  |
| YAP (phospho S127) | YAPPS127 | YAP1 | CST | 4911 | Rabbit | 1:750 |  |
| YB1 (phospho S102) | YB1PS102 | YBX1 | CST | 2900 | Rabbit | 1:50 |  |

**B. Overview of proteins and phospho-proteins used for calculation of pathway activation scores (Akbani *et al,* Nature Commun, 2014). Pathway members colored to aid visualisation.**

| **Pathway** | **Protein** | **Direction** | **Weight** |
| --- | --- | --- | --- |
| Apoptosis | BAK | + | 1 |
| Apoptosis | BAX | + | 1 |
| Apoptosis | BID | + | 1 |
| Apoptosis | BIM | + | 1 |
| Apoptosis | CASPASE7CLEAVEDD198 | + | 1 |
| Apoptosis | BADPS112 | - | 1 |
| Apoptosis | BCL2 | - | 1 |
| Apoptosis | BCLXL | - | 1 |
| Apoptosis | CIAP | - | 1 |
| Cell_cycle | CDK1 | + | 1 |
| Cell_cycle | CYCLINB1 | + | 1 |
| Cell_cycle | CYCLINE1 | + | 1 |
| Cell_cycle | P27PT198 | + | 1 |
| Cell_cycle | PCNA | + | 1 |
| Cell_cycle | FOXM1 | + | 1 |
| DNA_damage_response | 53BP1 | + | 1 |
| DNA_damage_response | ATM | + | 1 |
| DNA_damage_response | CHK2PT68 | + | 1 |
| DNA_damage_response | P53 | + | 1 |
| DNA_damage_response | RAD50 | + | 1 |
| DNA_damage_response | RAD51 | + | 1 |
| DNA_damage_response | XRCC1 | + | 1 |
| EMT | FIBRONECTIN | + | 1 |
| EMT | NCADHERIN | + | 1 |
| EMT | COLLAGENVI | + | 1 |
| EMT | CLAUDIN7 | - | 1 |
| EMT | ECADHERIN | - | 1 |
| EMT | PAI1 | + | 1 |
| Hormone_a | ER | + | 1 |
| Hormone_a | ERPS118 | + | 1 |
| Hormone_a | PR | + | 1 |
| Hormone_a | AR | + | 1 |
| Hormone_b | INPP4B | + | 1 |
| Hormone_b | GATA3 | + | 1 |
| Hormone_b | BCL2 | + | 1 |
| PI3K_AKT | AKTPS473 | + | 0.5 |
| PI3K_AKT | AKTPT308 | + | 0.5 |
| PI3K_AKT | GSK3ABPS21S9 | + | 1 |
| PI3K_AKT | P27PT198 | + | 1 |
| PI3K_AKT | PRAS40PT246 | + | 1 |
| PI3K_AKT | TUBERINPT1462 | + | 1 |
| PI3K_AKT | INPP4B | - | 1 |
| PI3K_AKT | PTEN | - | 1 |
| RAS_MAPK | CJUNPS73 | + | 1 |
| RAS_MAPK | CRAFPS338 | + | 1 |
| RAS_MAPK | JNKPT183Y185 | + | 1 |
| RAS_MAPK | MAPKPT202Y204 | + | 1 |
| RAS_MAPK | MEK1PS217S221 | + | 1 |
| RAS_MAPK | P38PT180Y182 | + | 1 |
| RAS_MAPK | YB1PS102 | + | 1 |
| RTK | EGFRPY1173 | + | 1 |
| RTK | HER2PY1248 | + | 1 |
| RTK | HER3PY1289 | + | 1 |
| RTK | SHCPY317 | + | 1 |
| RTK | SRCPY416 | + | 0.5 |
| RTK | SRCPY527 | + | 0.5 |
| TSC_mTOR | 4EBP1PS65 | + | 1 |
| TSC_mTOR | MTORPS2448 | + | 1 |
| TSC_mTOR | S6PS235S236 | + | 0.5 |
| TSC_mTOR | S6PS240S244 | + | 0.5 |
| TSC_mTOR | RBPS807S811 | + | 1 |
| Breast_reactive | CAVEOLIN1 | + | 1 |
| Breast_reactive | RAB11 | + | 1 |
| Breast_reactive | BCATENIN | - | 1 |
| Breast_reactive | GAPDH | - | 1 |
| Breast_reactive | RBM15 | - | 1 |
| Core_reactive | CAVEOLIN1 | + | 1 |
| Core_reactive | BCATENIN | - | 1 |
| Core_reactive | RBM15 | - | 1 |
| Core_reactive | ECADHERIN | - | 1 |
| Core_reactive | CLAUDIN7 | - | 1 |
